# Running gait modifications can lead to immediate reductions in patellofemoral pain

**DOI:** 10.3389/fspor.2022.1048655

**Published:** 2023-01-16

**Authors:** Jean-Francois Esculier, Laurent J. Bouyer, Jean-Sébastien Roy

**Affiliations:** ^1^ The Running Clinic, Lac beauport, QC, Canada; ^2^ Department of Physical Therapy, Faculty of Medicine, University of British Columbia, Vancouver, BC, Canada; ^3^ MoveMed Physiotherapy, Kelowna, BC, Canada; ^4^ Faculty of Medicine, Université Laval, Quebec, QC, Canada; ^5^ Centre for Interdisciplinary Research in Rehabilitation and Social Integration, Quebec, QC, Canada

**Keywords:** Gait retraining, Running, Step rate, Foot strike pattern, Knee pain

## Abstract

Gait modifications are commonly advocated to decrease knee forces and pain in runners with patellofemoral pain (PFP). However, it remains unknown if clinicians can expect immediate effects on symptoms. Our objectives were (1) to compare the immediate effects of gait modifications on pain and kinetics of runners with PFP; (2) to compare kinetic changes in responders and non-responders; and (3) to compare the effects between rearfoot strikers (RFS) and non-RFS. Sixty-eight runners with PFP (42 women, 26 men) ran normally on a treadmill before testing six modifications: 1- increase step rate by 10%; 2- 180 steps per minute; 3- decrease step rate by 10%; 4- forefoot striking; 5- heel striking; 6- running softer. Overall, there were more responders (pain decreased ≥1/10 compared with normal gait) during forefoot striking and increasing step rate by 10% (both 35%). Responders showed greater reductions in peak patellofemoral joint force than non-responders during all conditions except heel striking. When compared with non-RFS, RFS reduced peak patellofemoral joint force in a significant manner (*P *< 0.001) during forefoot striking (partial *η*^2^ = 0.452) and running softer (partial *η*^2^ = 0.302). Increasing step rate by 10% reduced peak patellofemoral joint force in both RFS and non-RFS. Forty-two percent of symptomatic runners reported immediate reductions in pain during ≥1 modification, and 28% had reduced pain during ≥3 modifications. Gait modifications leading to decreased patellofemoral joint forces may be associated with immediate pain reductions in runners with PFP. Other mechanisms may be involved, given that some runners reported decreased symptoms regardless of kinetic changes.

## 1. Introduction

Overuse injuries are frequent in runners (1), and patellofemoral pain (PFP) is among the leading diagnoses (2). Given that the patellofemoral joint (PFJ) is subject to high levels of compression forces during running (3), clinicians often recommend gait modifications to help alleviate symptoms of injured runners (4). These interventions include manipulating step rate (4–7) or foot strike pattern (4, 7, 8), and recommending to “run softer” (9–11).

Evidence suggests that gait modifications effectively modulate PFJ forces in healthy runners displaying a rearfoot strike pattern (RFS) (12). Increasing preferred step rate by 10% can reduce peak PFJ force by up to 14% (13), while decreasing step rate tends to increase peak PFJ force (14). A fixed step rate of 180 steps/min has also been recommended in the popular running literature to help reduce lower limb forces (15, 16), although its effect on PFJ kinetics has not been investigated. Transitioning to a forefoot strike pattern (17, 18) and “running softer” (9, 10, 19) have also been advocated to reduce lower limb forces (9, 10, 20), knee loading, and potentially reduce running-related injuries.

Unfortunately, research remains scarce on the effects of gait modifications in non-RFS runners, who are not immune to PFP (21). Given the considerable differences in kinematics and kinetics between RFS and non-RFS (22), it is likely that these subgroups react differently to gait modifications. For example, non-RFS tend to land with less impact than RFS, which could make it harder for these runners to “run softer”. In contrast, increasing step rate could potentially be more useful for non-RFS, given that their habitual step rate tends to be similar to that of RFS (22).

Pain modulation is the ultimate objective when retraining injured runners' gait. Previous studies, however, leave clinicians in the dark regarding the immediate effects of gait modifications when first implemented. Gait retraining programs typically involve multiple appointments. For example, training rearfoot strikers with PFP to transition to forefoot striking over 8 sessions can result in significant clinical improvements (7, 8, 23). Concurrent decreases in running-related pain and peak PFJ force following gait retraining (8) suggests an association between both variables (8). However, many other non-physical factors could be involved, such as sensory and emotional processing associated with the pain experience (24).

Without information on immediate effects, and potential mechanisms of action, a gap remains in the clinical uptake of gait retraining interventions. Indeed, immediate pain reduction has been suggested as a key priority to gain patient trust (25) and improve patient expectations. The absence of immediate effects could perhaps discourage a subset of patients, by lack of resources (e.g. time or financial constraints) or perceived benefits of altering gait mechanics.

The objectives of this study were (1) to compare the immediate effects of running gait modifications on lower limb kinetics and pain of runners with PFP; (2) to compare kinetic changes in immediate responders and non-responders; and (3) to compare the effects of gait modifications between RFS and non-RFS. We hypothesized that increasing step rate, forefoot striking and running softer would decrease PFJ forces in RFS runners, which would be associated with immediate reductions in pain during running. In non-RFS, we hypothesized that only increased step rate would have such effects.

## 2. Materials and methods

### 2.1. Population

A sample of 68 runners with PFP (Table 1) took part in this cross-sectional laboratory investigation, which was conducted during data collection for a randomised clinical trial (26). To be included, participants had to be aged between 18 and 45 years, run ≥15 km per week, present a history of PFP (pain originating from the patellofemoral joint) for ≥3 months, be comfortable running on a treadmill and report pain ≥3/10 on a visual analog scale during most of their running trainings and during three or more activities among: stairs, squatting, kneeling and resisted knee extension. Runners were excluded if they presented a history of lower limb surgery or patellar dislocation, pain believed to originate either from meniscus (27) or from patellar tendon (28), pain following an acute trauma, concurrent lower limb injuries or a history of neurological, inflammatory or rheumatoid disease. Runners were included regardless of foot strike pattern and footwear. Ethics approval was obtained from the Institutional Review Board (#2014-367). All participants signed a detailed consent form before entering the study.

**Table 1:** Characteristics of runners. Data presented as Mean (SD)

	All runners (*n = *68)	RFS (*n* = 45)	Non-RFS (*n = *23)
Gender (W/M)	42/26	30/15	12/11
Age (years)	30.7 (6.5)	30.3 (6.7)	31.3 (6.1)
Height (cm)	169.9 (9.0)	169.7 (9.4)	170.1 (8.5)
Mass (kg)	67.5 (14.2)	69.2 (15.6)	64.1 (10.4)
Weekly running distance (km)	20.3 (5.6)	19.5 (4.6)	22.0 (6.8)
Running experience (years)	6.1 (5.8)	6.3 (6.6)	5.7 (3.7)
Symptoms duration (months)	29.2 (39.0)	27.3 (38.1)	32.9 (41.3)
Worst knee pain during running (0–10)	6.0 (2.0)	5.7 (2.0)	6.5 (1.8)
Knee Outcome Survey – Activities of Daily Living Scale score (0–100)	70.4 (9.2)	69.5 (9.9)	72.1 (7.5)
Minimalist Index score (0–100)	36.1 (19.2)	30.5 (16.5)	47.1 (19.7)
Running speed (m/s)	2.5 (0.2)	2.5 (0.2)	2.5 (0.2)
Step rate (steps/min)	168.3 (10.3)	166.1 (9.2)	172.8 (11.0)

The Minimalist Index (0–100) is a scale used to quantify the level of minimalism in running shoes (a score of 100 represents highly minimalist shoes). Worst knee pain during running was assessed using a numeric pain rating scale (0 represents no pain, and 10 represents the worst pain imaginable). The Knee Outcome Survey – Activities of Daily Living Scale is a validated scale to assess the level of symptoms and function in individuals with PFP (a score of 100 represents the absence of symptoms and limitations).

### 2.2. Study design and experimental procedures

First, data on anthropometry, socio-demographics and symptoms were collected. Participants also completed the French version of the Activities of Daily Living Scale of the Knee Outcome Survey questionnaire (29) and a numerical pain rating scale for typical knee pain during running (0 = no pain, 10 = worst pain imaginable). Then, they were equipped with rigid clusters of spherical retroreflective markers placed on the lateral part of the feet, shanks and thighs, and at the lumbosacral and cervicothoracic regions (21). Thigh clusters were secured using Velcro straps to minimize movement artefacts induced by muscle contractions. For calibration purposes, anatomical markers were temporarily applied over the head of fifth metatarsals, anterior and posterior tips of the shoes, medial and lateral malleoli and femoral condyles, anterior superior iliac spines, iliac crests and lateral tip of the acromion.

Runners walked for at least one minute to ensure proper familiarization with the instrumented treadmill (Bertec corp, Columbus, OH). When they felt comfortable, treadmill speed was increased to self-selected running speed within the range of 8 to 10 km/h (2.3–2.7 m/s), to reproduce habitual training conditions. Following a five-minute warm-up period, kinematic and ground reaction force data were collected simultaneously for three minutes (condition: Normal) using an 8-camera Vicon MX-T system with Vicon Nexus software (Vicon motion systems, CA; sampling rate of 200 Hz) and the instrumented treadmill (sampling rate of 1,000 Hz).

After collecting data on habitual running pattern, runners were tested under six gait modifications: 1- step rate increased by 10% (SR + 10%); 2- step rate fixed at 180 steps per minute (SR180); 3- step rate decreased by 10% (SR-10%); 4- forefoot striking (FFS); 5- heel striking (HS); 6- running softer (SOFT). Half of runners followed this sequence, while the other half did it in reverse order. All runners performed all conditions; habitual RFS were asked not to exaggerate the HS condition and runners displaying a habitual non-RFS were asked not to exaggerate the FFS condition. To reproduce typical conditions from a clinical setting in which acute gait modifications are suggested to patients, participants were always provided with a minimum period of 30 seconds of habituation to each running modification before kinetic and kinematic data were collected during the ensuing minute. Data collection for each condition was initiated after visually ensuring that participants followed instructions, exhibited a regular running pattern, and reported feeling comfortable doing so. Immediately after each condition, runners verbally rated their average level of knee pain during the previous minute on a numerical pain rating scale of 0 to 10, where 0 meant “no pain” and 10 meant the “worst imaginable pain”. A wash-out period of at least one minute (return to habitual running pattern) was used between gait modifications to avoid carryover effects. Before explaining the next gait modification, an experienced physical therapist ensured that runners returned to baseline step rate, foot strike pattern and pain levels if decreased pain was reported during the previous condition. Auditory cues for step rate modifications were provided using a metronome (5). Verbal feedback was provided to runners about the different gait modifications. Total running duration did not exceed 30 minutes. All runners wore their habitual running shoes during testing, which were rated using the Minimalist Index (30).

### 2.3. Outcome measures and data analysis

All runners (*n* = 68) were considered for the analysis of biomechanical outcomes. Variables of interest included peak PFJ force and average loading rate, peak Achilles tendon force, as well as the vertical ground reaction force's average loading rate during the stance phase of each condition. We included Achilles tendon force to detect if lower limb kinetics were reduced or shifted distally with gait modifications. As for symptoms, only runners reporting knee pain of at least 1/10 during the Normal condition at time of testing (*n* = 43) were further classified for each gait modification into responders (when pain level decreased by ≥1/10 compared with the Normal condition). Non-responders were further described as stable or reporting increased pain (≥1/10 compared with the Normal condition). Such threshold was chosen to capture mild immediate changes in knee pain, as they would be reported in clinical conditions, to reflect potential perceived benefits from injured runners.

Data were analyzed using custom-written MatLab programs (Mathworks Inc., MA). Marker trajectory data were processed through a sagittal, frontal, transverse cardan rotation sequence to extract lower-limb sagittal plane angles. Kinematics and ground reaction force data were filtered using zero-lag fourth-order low-pass 12 Hz and 30 Hz Butterworth filters, respectively. Initial contact and toe-off were determined using a 20 N threshold. The average of fifty symptomatic limb stance phases during each condition (collected following the habituation period) was considered for data analysis to account for possible variations in mechanics associated with unfamiliar running patterns. Then, Newton-Euler inverse dynamics equations were used to estimate the shear and compression reaction forces at the knee and ankle joints. PFJ reaction force was estimated using a previously reported algorithm (31) that considers knee flexion angle and net knee extension moment along with quadriceps moment arm reported by van Eijden et al. (32). To calculate PFJ average loading rate, the 20% and 80% points between initial rise in force and the first force peak were identified. Differences in force values between these two points were divided by the number of time points to obtain loading rate (33). Vertical ground reaction force's average loading rate was obtained using the same procedures (34). Achilles tendon forces were estimated as a function of the net plantar flexion moment relative to the Achilles tendon moment arm, which was derived from the ankle flexion angle (35). Classification into RFS and non-RFS subgroups was made using video-based inspection of foot strike pattern, which has been shown to be valid and reliable in runners with PFP (36).

### 2.4. Statistical analyses

For Objective 1, mean values for pain and kinetic variables of interest were compared across conditions (*n* = 7) using generalized repeated-measures ANOVAs (generalized estimating equations; distribution = normal; *P*-values corrected by sequential Bonferroni). For Objectives 2 and 3, kinetic changes for each condition were compared between responders and non-responders, and RFS and non-RFS, respectively, using repeated-measures ANOVAs. Effect sizes (partial *η*^2^) were calculated, and considered small when ≥0.01, moderate when ≥0.06 or large when ≥0.14 (37). Statistical analyses were performed using SPSS 27.0 (SPSS Inc., IL). The level of statistical significance was set at *P* = 0.05, and 95% confidence intervals were reported for all variables of interest.

## 3. Results

### 3.1. Immediate effects of gait modifications on pain and kinetics

**Figure 1:**
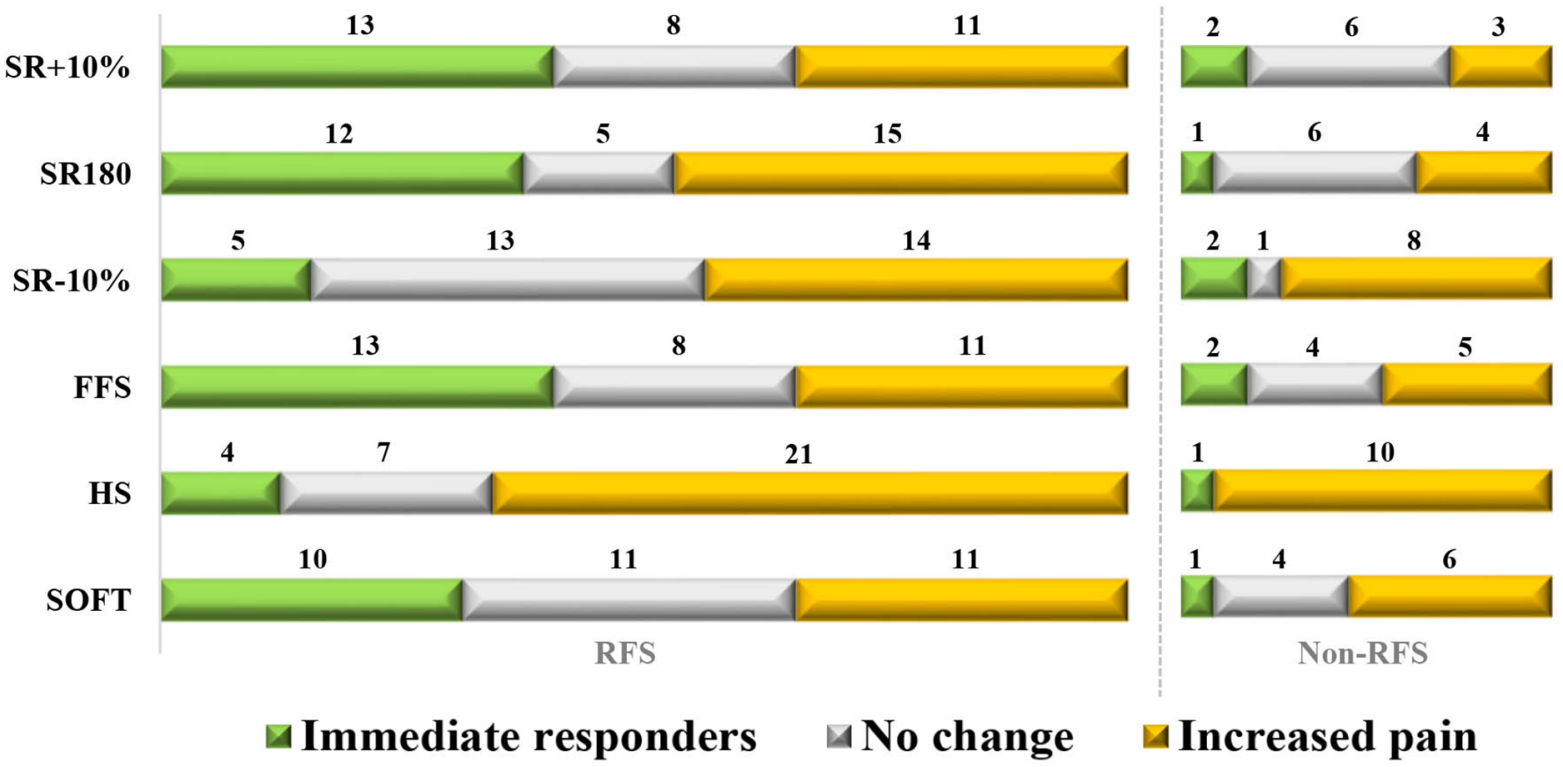
Number of responders to each gait modification for RFS (*n* = 32) and non-RFS (*n* = 11). Responders were defined as runners reporting a decrease of ≥1/10 of their pain level compared with the Normal condition. “Increased pain” means a pain increase of ≥1/10 compared with the Normal condition.

A total of 43 runners (32 RFS, 11 non-RFS) experienced pain >1/10 during the Normal condition, and 20 of them (47%) were classified as responders during at least one gait modification. The most effective interventions were FFS and SR + 10% (15 responders each), but some runners also reported decreased pain during SR-10% (7 responders) and HS (5 responders) (Figure 1). Some participants were responders to three (*n* = 2), four (*n* = 5), five (*n* = 3) and even all six gait modifications (*n* = 2). Each gait modification also caused an increase in pain in a subset of runners, with proportions varying between 33% (SR + 10%, *n* = 14) and 72% (HS, *n* = 31) (Figure 1). Detailed pain scores during each condition are provided in Supplemental Material.

Overall (*n* = 68), SR + 10% and FFS showed the greatest reduction in peak PFJ force (both −12%), followed by SOFT (-11%) (Table 2). PFJ average loading rate was significantly decreased during FFS (−32%), SOFT (−29%) and SR-10% (−10%) (Table 2). Statistically significant reductions in the average vertical loading rate of the ground reaction force were achieved only through FFS (−42%) and SOFT (−31%) (Table 2). Both FFS (+11%) and SR-10% (+4%) increased peak Achilles tendon force, while HS was the only condition causing a statistically significant decrease (−12%) (Table 2).

**Table 2:** Comparisons of kinetic variables between Normal and the different gait modifications (*n* = 68), presented as Mean [95% C.I.]. *P*-values relate to differences with the Normal condition.

	Peak PFJ force (BW)	*P*	PFJ average loading rate (BW/s)	*P*	Average vertical loading rate (BW/s)	*P*	Peak Achilles tendon force (BW/s)	*P*
Normal	3.4 [3.2, 3.5]		63.6 [58.8, 68.5]		39.5 [35.4, 43.6]		4.7 [4.5, 4.8]	
SR + 10%	3.0 [2.8, 3.2]^a^	<0.001	58.6 [53.0, 64.1]	0.161	35.2 [31.4, 38.9]	0.066	4.6 [4.5, 4.8]	1.000
SR180	3.2 [3.0, 3.4]	0.160	59.4 [54.4, 64.4]	0.373	36.4 [32.6, 40.3]	0.097	4.7 [4.4, 4.9]	1.000
SR-10%	3.6 [3.4, 3.7]^b^	<0.001	57.5 [52.7, 62.4]^a^	0.017	41.5 [37.0, 46.1]	0.333	4.8 [4.7, 5.0]^b^	<0.001
FFS	3.0 [2.8, 3.1]^a^	<0.001	43.4 [38.7, 48.0]^a^	<0.001	23.1 [21.7, 24.5]^a^	<0.001	5.2 [5.1, 5.3]^b^	<0.001
HS	3.7 [3.6, 3.9]^b^	<0.001	67.6 [63.7, 71.4]	0.373	45.1 [40.5, 49.6]	0.097	4.1 [4.0, 4.2]^a^	<0.001
SOFT	3.0 [2.8, 3.2]^a^	<0.001	45.0 [40.1, 49.9]^a^	<0.001	27.2 [24.0, 30.3]^a^	<0.001	4.4 [4.2, 4.6]	0.132

SR + 10%, Step rate increased by 10%; SR180, Step rate of 180 steps per minute; SR-10%, Step rate decreased by 10%; FFS, Forefoot strike; HS, Heel strike; SOFT, Running softer.

^a^ means a statistically significant decrease compared with Normal.

^b^ means a statistically significant increase compared with Normal.

### 3.2. Differences between responders and non-responders

Responders experienced significantly greater reductions in peak PFJ force than non-responders (partial *η*^2^ = 0.064 to 0.106, moderate effect sizes) during all conditions except HS (Table 3A). However, no statistically significant differences in PFJ average loading rate or in the average loading rate of the ground reaction force were found between responders and non-responders (Table 3A). Responders significantly increased Achilles tendon peak force compared to non-responders only during SR + 10% (partial *η*^2^ = 0.088, moderate effect size) (Table 3A).

**Table 3:** Comparisons of changes in kinetic variables (**A**) between immediate responders and non-responders to gait modifications, and (**B**) between RFS and non-RFS. Data presented as Mean difference with Normal [95% C.I.], and *P*-value and effect size for the interaction between mean difference * gait modification.

(A) Responders vs. non-responders (*n* = 43)						
	SR + 10%	SR180	SR-10%	FFS	HS	SOFT
Peak PFJ force (BW)						
Responders	−0.7 [−1.0, −0.4]	−0.6 [−0.9, −0.2]	−0.1 [−0.4, 0.2]	−0.6 [−0.8, −0.4]	0.3 [−0.2, 0.9]	−0.6 [−0.8, −0.4]
Non-responders	−0.3 [−0.4, −0.2]	−0.1 [−0.3, 0.1]	0.2 [0.1, 0.3]	−0.3 [−0.4, −0.2]	0.4 [0.2, 0.5]	−0.3 [−0.4, −0.1]
	*P* = 0.008	*P* = 0.029	*P* = 0.037	*P* = 0.008	*P* = 0.865	*P* = 0.007
	Partial *η*^2^ = 0.102	Partial *η*^2^ = 0.070	Partial *η*^2^ = 0.064	Partial *η*^2^ = 0.101	Partial *η*^2^ = 0.0	Partial *η*^2^ = 0.106
PFJ average loading rate (BW/s)						
Responders	−8.2 [−18.1, 1.8]	−10.1 [−19.8, −0.5]	−8.2 [−18.3, 1.9]	−20.8 [−28.8, −12.8]	2.1 [−9.4, 13.6]	−18.6 [−25.0, −12.2]
Non-responders	−4.2 [−9.2, 0.7]	−2.9 [−7.9, 2.2]	−5.9 [−10.1, −1.7]	−20.1 [−26.0, −14.3]	4.1 [−0.5, 8.6]	−17.1 [−21.8, −12.4]
	*P* = 0.474	*P* = 0.218	*P* = 0.730	*P* = 0.912	*P* = 0.812	*P* = 0.782
	Partial *η*^2^ = 0.008	Partial *η*^2^ = 0.023	Partial *η*^2^ = 0.002	Partial *η*^2^ = 0.0	Partial *η*^2^ = 0.001	Partial *η*^2^ = 0.001
Average vertical loading rate (BW/s)						
Responders	−9.0 [−16.3, −1.7]	−8.9 [−15.2, −2.5]	−4.1 [−14.9, 6.7]	−23.4 [−30.0, −16.7]	7.8 [−11.3, 26.9]	−17.2 [−24.0, −10.4]
Non-responders	−3.2 [−7.0, 0.5]	−1.9 [−4.9, 1.0]	2.8 [0.3, 5.3]	−14.9 [−19.7, −10.0]	5.2 [0.4, 10.0]	−13.4 [−14.9, −7.9]
	*P* = 0.173	*P* = 0.054	*P* = 0.096	*P* = 0.100	*P* = 0.771	*P* = 0.221
	Partial *η*^2^ = 0.028	Partial *η*^2^ = 0.056	Partial *η*^2^ = 0.042	Partial *η*^2^ = 0.041	Partial *η*^2^ = 0.001	Partial *η*^2^ = 0.023
Peak Achilles tendon force (BW)						
Responders	0.2 [−0.1, 0.5]	0.1 [−0.2, 0.4]	0.2 [0.0, 0.4]	0.6 [0.4, 0.7]	−0.5 [−0.8, −0.1]	−0.2 [−0.4, 0.1]
Non-responders	−0.1 [−0.2, 0.0]	0.0 [−0.2, 0.2]	0.2 [0.1, 0.3]	0.5 [0.4, 0.6]	−0.6 [−0.7, −0.5]	−0.2 [−0.3, −0.1]
	*P* = 0.014	*P* = 0.637	*P* = 0.736	*P* = 0.582	*P* = 0.522	*P* = 0.307
	Partial *η*^2^ = 0.088	Partial *η*^2^ = 0.003	Partial *η*^2^ = 0.002	Partial *η*^2^ = 0.005	Partial *η*^2^ = 0.006	Partial *η*^2^ = 0.016
(B) RFS vs. non-RFS (*n* = 68)						
	SR + 10%	SR180	SR-10%	FFS	HS	SOFT
Peak PFJ force (BW)						
RFS	−0.3 [−0.5, −0.2]	−0.2 [−0.3, −0.1]	0.1 [0.0, 0.1]	−0.6 [−0.6, −0.5]	0.1 [0.1, 0.2]	−0.5 [−0.6, −0.4]
Non-RFS	−0.5 [−0.7, −0.4]	−0.1 [−0.6, 0.3]	0.4 [0.2, 0.6]	−0.1 [−0.2, 0.0]	0.8 [0.6, 1.0]	0.0 [−0.1, 0.2]
	*P* = 0.109	*P* = 0.651	*P* < 0.001	*P* < 0.001	*P* < 0.001	*P* < 0.001
	Partial *η*^2^ = 0.039	Partial *η*^2^ = 0.003	Partial *η*^2^ = 0.205^a^	Partial *η*^2^ = 0.452^a^	Partial *η*^2^ = 0.469^a^	Partial *η*^2^ = 0.302^a^
PFJ average loading rate (BW/s)						
RFS	−1.6 [−6.5, 3.3]	−1.5 [−7.4, 4.5]	−7.3 [−11.5, −3.1]	−22.8 [−29.1, −16.5]	−0.2 [−5.2, 4.9]	−17.8 [−22.8, −12.9]
Non-RFS	−11.9 [−20.2, −3.5]	−9.8 [−15.8, −3.7]	−3.8 [−11.8, 4.1]	−15.3 [−22.4, −8.3]	11.9 [5.0, 18.8]	−16.3 [−23.4, −9.2]
	*P* = 0.030	*P* = 0.088	*P* = 0.409	*P* = 0.912	*P* = 0.008	*P* = 0.734
	Partial *η*^2^ = 0.069	Partial *η*^2^ = 0.044	Partial *η*^2^ = 0.010	Partial *η*^2^ = 0.0	Partial *η*^2^ = 0.102	Partial *η*^2^ = 0.002
Average vertical loading rate (BW/s)						
RFS	−6.9 [−11.1, −2.6]	−6.0 [−9.8, −2.2]	3.1 [−0.5, 6.6]	−23.3 [−28.0, −18.5]	−3.2 [−6.7, 0.3]	−15.6 [−19.5, −11.8]
Non-RFS	0.2 [−4.6, 5.1]	2.1 [0.9, 3.4]	0.2 [−1.9, 2.3]	−3.9 [−8.2, 0.3]	21.7 [13.4, 30.0]	−5.7 [−10.0, −1.4]
	*P* = 0.048	*P* = 0.005	*P* = 0.287	*P* < 0.001	*P* < 0.001	*P* = 0.003
	Partial *η*^2^ = 0.059	Partial *η*^2^ = 0.117	Partial *η*^2^ = 0.017	Partial *η*^2^ = 0.290^a^	Partial *η*^2^ = 0.380^a^	Partial *η*^2^ = 0.132
Peak Achilles tendon force (BW)						
RFS	−0.1 [−0.2, 0.0]	0.0 [−0.2, 0.1]	0.1 [0.1, 0.2]	0.7 [0.6, 0.8]	−0.4 [−0.5, −0.3]	−0.1 [−0.2, 0.0]
Non-RFS	−0.1 [−0.2, 0.1]	0.2 [−0.3, 0.6]	0.2 [0.1, 0.4]	0.2 [0.1, 0.3]	−0.9 [−1.1, −0.7]	−0.3 [−0.5, −0.1]
	*P* = 0.535	*P* = 0.289	*P* = 0.243	*P* < 0.001	*P* < 0.001	*P* = 0.142
	Partial *η*^2^ = 0.006	Partial *η*^2^ = 0.017	Partial *η*^2^ = 0.021	Partial *η*^2^ = 0.360^a^	Partial *η*^2^ = 0.319^a^	Partial *η*^2^ = 0.033

*S*R + 10%, Step rate increased by 10%; SR180, Step rate of 180 steps per minute; SR-10%, Step rate decreased by 10%; FFS, Forefoot strike; HS, Heel strike; SOFT, Running softer; RFS, Rearfoot strikers; BW, Bodyweight.

^a^ denotes large effect sizes.

### 3.3. Differences between RFS and non-RFS

Proportions of immediate responders among RFS and non-RFS were 41% and 18% during both SR + 10% and FFS, 38% and 9% during SR180, and 31% and 9% during SOFT (Figure 1). The HS condition caused immediate increases in pain in 66% of RFS and 91% of non-RFS (Figure 1).

Changes in PFJ kinetics were statistically different between RFS and non-RFS. Specifically, RFS experienced a greater decrease in peak PFJ force during FFS and SOFT (partial *η*^2^ = 0.302–0.452, large effects) (Table 3B). Non-RFS increased peak PFJ force more than RFS during SR-10% and HS (partial *η*^2^ = 0.205–0.469, large effects). Non-RFS also had greater decreases in PFJ average loading rate during SR + 10% (partial *η*^2^ = 0.069, moderate effect) and SR180 (partial *η*^2^ = 0.044, small effect), but had an increase during HS (partial *η*^2^ = 0.102, moderate effect) (Table 3B).

The average loading rate of the ground reaction decreased in RFS more so than in non-RFS during FFS (partial *η*^2^ = 0.290, large effect), SOFT (partial *η*^2^ = 0.132, moderate effect), SR180 (partial *η*^2^ = 0.117, moderate effect), and SR + 10% (partial *η*^2^ = 0.059, small effect) (Table 3B). Non-RFS increased their average loading rate of the ground reaction force (partial *η*^2^ = 0.380, large effect) but decreased peak Achilles tendon force during HS more than RFS (partial *η*^2^ = 0.319, large effect). RFS experienced a significantly greater increase in peak Achilles tendon force during FFS (partial *η*^2^ = 0.360, large effect) (Table 3B).

## 4. Discussion

Increasing step rate, adopting a forefoot strike pattern and running softer may cause immediate reductions in pain in some runners with PFP, especially those using an RFS pattern. Decreased peak PFJ force could explain such effects, and potentially represents a key outcome during rehabilitation. For runners using a non-RFS pattern, SR + 10% might also be an option.

Immediate reductions in symptoms were reported by 75.0% (15 out of 20) of all responders during FFS and SR + 10%. Although several other studies have reported reductions in knee joint loading through gait modifications (5, 13, 14, 38, 39), the current study is the first to report concurrent immediate reductions in running-related knee pain. Switching to a FFS has been suggested as an effective treatment in runners with PFP (8, 23), although it is unknown whether runners in these studies experienced immediate or gradual decrease in knee pain. One of these studies found a reduction of both the peak PFJ contact force and symptoms in RFS runners (8), an association that was also present in our study. According to our comparison between immediate responders and non-responders, decreasing peak PFJ force seems highly relevant to reduce pain. The fact that 72% of symptomatic runners, including 91% of non-RFS, reported increased pain during HS also supports this hypothesis, given the overall increase in peak PFJ force during that condition.

It is possible that non-responders would have reported pain improvements following multiple sessions of gait retraining. Some runners may need more practice, and more feedback to effectively change their running gait, (40) and potentially affect symptoms. Decreasing peak PFJ force or modulating patellofemoral contact area represent sensible clinical targets, however, caution is warranted if deciding to implement FFS. Given the increased peak Achilles tendon force during FFS (41), gradual transition is needed to minimize the risks of developing an injury to the ankle plantar flexors. In contrast, increasing step rate may represent a safer gait modification, at least in the short term, given its negligible effect on peak Achilles tendon force and effectiveness in reducing peak patellofemoral joint force. Runners with PFP might benefit more from an increase in their baseline step rate (+10%) rather than aiming for a fixed value (e.g. 180 steps per minute), no matter which foot strike pattern they are using. Increasing step rate by 10% in our sample of non-RFS led to an average of 189 steps per minute, effectively reducing peak patellofemoral joint force. In contrast, 180 steps per minute corresponded to an increase of only 4.2%, and no significant change in peak patellofemoral force. However, based on the low number of immediate responders to SR + 10% in non-RFS, education and appropriate management of training loads should likely be prioritized over gait modifications in these runners (26).

Gait modifications may affect symptoms because of reasons unrelated to biomechanical changes. Interestingly, several of our participants reported positive effects on pain during multiple gait modifications – even those that tend to increase knee loading. This could possibly result from gait modifications acting on psychological features, an aspect of PFP that is garnering more interest (24). It has been suggested that apparently similar but motivationally different movement patterns, for example related to the expectation of treatment effects, may reduce movement-related pain (42). Thus, it is possible that gait modifications induce analgesic effects that are unrelated to mechanical loading (sensory), but rather to more complex (central) pain mechanisms in a subset of symptomatic runners, like those five participants who responded to five or even six different movement patterns.

Given the high number of non-responders in our cohort, factors other than a transient decrease in joint loading are likely to contribute to modulation of symptoms in runners with chronic PFP. Potential factors include sensitization secondary to long-standing pain (43), which could possibly delay beneficial effects of decreased loading. Furthermore, location of symptoms (retropatellar vs. peripatellar) may correspond to different physiopathology in runners with PFP, thereby influencing pain response to retropatellar loading modulation (44). On the other hand, several runners experienced increases in pain with gait modifications, despite reductions in peak PFJ force. It is possible that these participants increased the amount of muscle co-contractions around the patellofemoral joint, thereby increasing forces that were not captured by our biomechanical model. In such cases, clinicians should perhaps focus on interventions unrelated to running gait modifications, like education on training loads and exercises (26). More research is needed to better identify responders to gait modifications, and to see if immediate changes in pain may predict clinical success following prolonged exposure.

This study has limitations, which should be considered when interpreting the results. First, data were collected following a short period of adaptation to gait modifications. It remains unknown if runners would maintain the same running mechanics following prolonged periods, and if changes in symptoms would last. Still, our results show that clinicians can expect acute gait modifications to immediately affect the level of knee pain of runners with PFP, and potentially get patients to perceive their potential benefits. Running gait modifications can sometimes be used as a temporary measure to modulate load, and not always as a permanent intervention, just like reductions of training loads can provide pain relief before increasing back to baseline level when pain allows (26). Second, the accumulation of different running conditions within the same session may have prevented modulations in loading from having beneficial effects on pain. However, total running duration did not exceed 30 minutes, which was easily reached by participants during their habitual training sessions. Third, the order of conditions was not randomized, but rather pre-determined because of logistical reasons related to the laboratory setup. Although not as optimal as a randomized order, we believe that reversing the order for the second half of participants helped in limiting potential biases. Fourth, we classified responders based on pain changes of at least 1/10. Despite being inferior to the meaningful pain changes in populations with chronic conditions (45), our study design allowed for immediate assessment of pain with negligible recall bias. Fifth, our musculoskeletal model did not take into account muscle co-activations, and thus might have underestimated PFJ forces. Even though kinetics comparisons between conditions may have been affected, our within-subject design minimizes negative implications of using such model. Finally, the number of runners using a non-RFS pattern was relatively low. However, the proportion of runners using a non-RFS pattern is only 21% (46). Therefore, our sample is representative of the running population, and our findings may help orientate future research in this sub-group of runners with PFP.

In conclusion, results from this study suggest that gait modifications can immediately affect the level of symptoms in runners with PFP, likely because of modulations in peak PFJ force. Specifically, FFS and increasing step rate by 10% were the most effective in reducing pain and PFJ kinetics, with greater effects in runners displaying an RFS during their normal running. Instructions to run softer were also effective in RFS, but may not be as effective in non-RFS. Significant decreases in peak PFJ force were achieved when increasing step rate by 10%, running at 180 steps per minute, landing on the forefoot and landing softer in RFS, but only when increasing step rate by 10% in non-RFS. Decreases in peak PFJ force could represent a key rehabilitation target, considering the number of runners reporting decreased pain during these gait modifications. However, our results suggest that the immediate effects of gait modifications on pain may not solely be attributable to modulations of PFJ kinetics. More research is needed to investigate the effects of prolonged exposure to gait modifications in immediate responders and non-responders, and to evaluate whether clinical benefits of gait modifications could be predicted by such immediate response.

## Data Availability

The raw data supporting the conclusions of this article will be made available by the authors, without undue reservation.
