# Supplementary material for: Running gait modifications can lead to immediate reductions in patellofemoral pain
Source: Front Sports Act Living. 2023 Jan 16;4:1048655. doi: 10.3389/fspor.2022.1048655 (PMC9884822; doi:10.3389/fspor.2022.1048655)
Supplement: Supplementary file 1 [file Datasheet1.pdf]

Supplementary File 1: Pain scores during each condition for each participant, where 0 means "No pain" and 10 means "Worst pain imaginable".

	NORMAL	SR+10%	SR180	SR-10%	FFS	HS	SOFT
PFP01	4	2	0	0	0	0	0
PFP02	0	1.5	2	0	5	3	6
PFP03	0	0	0	0	0	0	4
PFP04	0	2	2	2	2	3	2
PFP05	1.5	1.5	3	0	0	0	0
PFP06	5.5	4	0	6	5	7	5
PFP07	0	0	0	0	0	0	0
PFP08	2	1	1	3	1	3	2
PFP09	0	0	0	0	1	2	2.5
PFP10	2	0	0	0	0	0	0
PFP11	0	0	0	0	0	0	0
PFP12	0	0	0	0	0	0	0
PFP13	0	0	0	0	0	0	0
PFP14	0	1	1	3	0	0	4
PFP15	2.5	5.5	5	3	3	5.5	6
PFP16	0	0	0	0	0	0	0
PFP17	3	2	3.5	1	2.5	4.5	5
PFP18	0	0	0	0	3.5	1	0
PFP19	2	2	2	3	2	3	2
PFP20	0	0	0	1	0	0	0
PFP21	0	1	1	1.5	0	1.5	1
PFP22	0	3	2.5	2.5	0	3	0
PFP23	0	1	0	0	0	0	0
PFP24	0	2.5	0	0	0	3	0
PFP25	2	0	0	1	0.5	4	1
PFP26	1.5	0.5	0.5	1	1	1	1.5
PFP27	2	3	3	2	0	3	2
PFP28	3	3	4	5	4	5	3
PFP29	1	1	1	1	2	3	2
PFP30	2.5	5.5	4	3	3	3.5	2.5
PFP31	0	4.5	2	4	4	3	0
PFP32	0	0	0	0	0	0	0
PFP33	3	5	5	4.5	4.5	4	5
PFP34	0	2	2	5	0	4	3
PFP35	0	1	2	1	1	1	0
PFP36	2	3	3	3	3	3	3
PFP37	2	4.5	4	2.5	3	4	4
PFP38	6	7.5	7	6	6	6	5
PFP39	2	0	0	2	0	2	0
PFP40	4	6	5	5	5	5	5
PFP41	2	3	3	5	3	2	4
PFP42	5	4	4	6	1	6	5
PFP43	3	4	4	4	4	5	2

PFP44	4	3.5	4.5	6	5.5	5	4
PFP45	2	3.5	2.5	2.5	1	3	2
PFP46	1	3.5	3.5	3	3	3.5	2
PFP47	0	1	0	2	0	0	0
PFP48	0	2.5	2	1	1	1	1
PFP49	2	0	1	0	0	4	0
PFP50	1	2	2	3	4	4	2
PFP51	6.5	2	3	3	4	5	6.5
PFP52	4	4	4	4	4	5	4
PFP53	0	0	0	0	0	0	0
PFP54	0	2	1	1	1	2	0
PFP55	3	2	4	4	0	2	3
PFP56	2	2	2	3	2.5	4	3
PFP58	2.5	1	0	2	1	4	1
PFP59	2	5	7	6	4	6	5
PFP60	3	3	3	4	6	4	4
PFP61	5.5	5.5	6.5	6.5	5	6.5	7.5
PFP62	2	2	4	5	5	5.5	5.5
PFP63	4	4	4.5	5.5	4	5	4.5
PFP64	1.5	1	1.5	1.5	1	2	1
PFP65	4.5	3	2.5	7	1	5	2.5
PFP66	7.5	6.5	6	8	6	7	6
PFP67	3	3.5	5	5.5	5.5	4	6
PFP68	0	1	1	2	2	3	3
PFP69	4	4	4.5	5	5	5	6
